# Investigation of potentially pathogenic *Vibrionaceae* in Saint-Louis city, Senegal

**DOI:** 10.11604/pamj.2024.48.5.34685

**Published:** 2024-05-03

**Authors:** Seynabou Lo, Bissoume Sambe Ba, Aissatou Ahmet Niang, Issa N'diaye, Mamadou Diop, Guillaume Constantin De Magny

**Affiliations:** 1 UFR Sciences of Health, Gaston Berger University, Saint-Louis, Senegal,; 2 Laboratory of Biology, Regional Hospital Center of Saint-Louis, Senegal,; 3 Pole of Microbiology, Pasteur Institute, Dakar, Senegal,; 4 Laboratory of Bacteriology and Virology, FMPOS, UCAD, Dakar, Senegal,; 5 Laboratory of Bacteriology and Virology, Fann National University Hospital Center, Dakar, Senegal,; 6 Montpellier Ecology and Evolution of Disease Network (MEEDiN), Montpellier, France,; 7 MIVEGEC (Université de Montpellier, UMR CNRS 5290, IRD 224), Institut de Recherche pour le Développement Délégation Occitanie, Montpellier, France

**Keywords:** Infectious disease, clinical, environment

## Abstract

**Introduction:**

as cholera, due to toxigenic bacteria Vibrio cholera (serogroups O1 and O139), is a major public health threat in Africa, the aim of this work was to investigate potentially pathogenic Vibrionaceae bacteria firstly from human stool samples, and secondly from various environmental water points of Saint-Louis city in Senegal.

**Methods:**

a hospital-based study was conducted between 2013 and 2015. Stool samples were taken and cultured from daily incoming patients or hospitalized for acute diarrhea at Saint-Louis´ regional hospital. For environment, a monthly longitudinal sampling from January to October 2016 was carried out at 10 sites in the city. We used total DNA extracted from APW (alkaline peptone water) broth solutions and on suspect bacterial colonies to run PCR Multiplex targeting specific DNA fragments to detect Vibrio genus and specific species. In case of positivity, a simplex PCR was performed to test for cholera toxins Ctx, and V. parahaemolyticus TRH and TDH.

**Results:**

for 43 patients screened, bacterial culture was positive in 6% of cases but no strain of V. cholerae or other Vibrio sp. was isolated. PCR on 90 APW solutions were positive for Vibrio sp.(n = 43), V. cholera(n = 27), V. mimicus(n = 16), V. parahaemolyticus(8), V. alginolyticus(n = 4), and V. vulnificus(n = 2). Unlike for those on suspected colonies which were positive for a majority of V. parahaemolyticus (n = 40) and V. cholerae non-O1 / O139 (n = 35). Six strains of V. parahaemolyticus carried TRH gene, 3 of which expressed simultaneously virulence TRH and TDH genes. For physicochemical parameters, all temperatures varied similarly according to a unimodal seasonality, as well as salinity.

**Conclusion:**

despite the presence of natural populations of Vibrionaceae, even toxigenic ones, was noted in water environment, along with favorable habitat conditions that could play a role in transmission of Vibriosis in the Saint Louis population, we did not isolate any of them from patients screened at the hospital.

## Introduction

Among the bacteria of Vibrio genus present in marine and estuarine environments, three are human pathogens, i.e. toxigenic *V. cholerae* O1 and O139 causing cholera, *V. parahaemolyticus* TDH and/or TRH positives, and *V. vulnificus* [1]. Several other species of Vibrio genus, such as *V. mimicus* and *V. alginolyticus* are also potentially pathogenic for humans [2]. These species had been isolated in many coastal waters around the world [3-8]. Some species like *V. vulnificus* can be responsible for serious infections in people with immunodeficiency and can evolve quickly into sepsis. This species was found in coastal and brackish waters (with variable salinity) such as estuaries and lagoons [9]. About *Vibrio cholerae*, many serotypes of non-O1 and non-139 occur naturally within the marine and estuarine ecosystems where they play an important ecological role and can also sporadically be responsible for gastroenteritis in humans [1]. Human toxigenic strains of *Vibrio cholera* serogroups O1 or O139, responsible for cholera can be isolated from aquatic environment almost entirely during ongoing cholera outbreak [10,11]. Ecological dynamics of Vibrio spp. in the aquatic ecosystem are directly influenced by environmental and climatic conditions [1]. These parameters have an impact on their presence, persistence and abundance in the aquatic ecosystem [12].

Senegal suffered from several cholera outbreaks since seventh pandemic in 1971, with the last epidemic in 2004-2005 that caused 31.719 cases and 458 deaths, and more specifically Saint Louis city recorded 1.185 cases [13]. The city of Saint-Louis in Senegal offers a specific and favorable environment of *Vibrio genus* because it has simultaneous presence of fresh water (Senegal river), brackish water (Senegal river estuary) and marine water (Atlantic Ocean). Indeed, the city is located in the northwest of Senegal at the mouth of Senegal river and is an integral part of Ferlo area with its Sahelian climate. Predominant human activities are fishing and agriculture as result of permanent contact with waters and consumption of fishery products. Consequently, there is a significant risk of emergence and transmission of Vibriosis in Saint-Louis. The objective of this study was to conduct a systematic search for potentially pathogenic Vibrionaceae species according to two research axes: (i) active surveillance of *Vibrios* in stools of patients seen on an outpatient basis or admitted to the Saint-Louis regional hospital for diarrhoea, (ii) *Vibrios* presence was examined in the surrounding aquatic environment combined to the measure of physico-chemical parameters favorable to their development and detection. In current medical practice, detection for toxigenic *Vibrio cholerae* in human was done only when there was a suspicion of cholera or during an ongoing epidemic. This work took place over a one-year period covering all season (pre-winter, winter and post-winter) in this region where there is permanent contact of population with water, and deliberately not during the course of an outbreak.

## Methods

**Study design:** a hospital-based study was conducted to evaluate the prevalence of vibriosis among daily incoming patients or hospitalized for acute diarrhea at Saint-Louis´s regional hospital from stool samples between 2013 and 2015. Thereafter, Vibrios presence was examined in the surrounding aquatic environment of Saint-Louis combined to the measure of physico-chemical parameters favorable to their development and detection during the year 2016.

**Figure 1:**
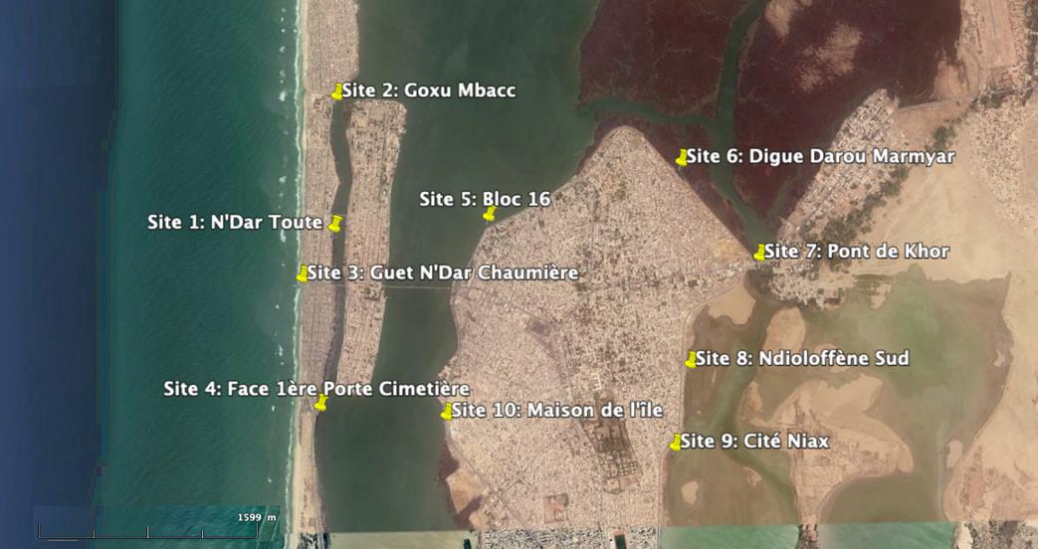
mapping of ten sites sampled for environmental monitoring of *Vibrio sp*. in Saint-Louis city, Senegal

**Data collection:** stool samples were taken from patient with diarrhoea (outpatient or hospitalized) between March 13^th^, 2013 and April 15^th^, 2015. Culture was carried out according to the procedure adopted by the laboratory by testing for *Vibrio cholera* on thiosulfate-citrate-bile salts-sucrose (TCBS) agar and ChromAgar *Vibrio, Salmonella* and *Shigella* on selective Salmonella Shigella (SS) agar. In children under 5, in addition to these pathogens, *E. coli* was researched on eosin and methylene blue agar and Adenovirus/Rotavirus by agglutination of latex particles sensitized with corresponding monoclonal antibodies. Focusing on only hospital-based patient allow to focus on most severe cases but may be a limitation representing bias on the representativeness of patients suffering from diarrhea in Saint-Louis area. For water sampling, longitudinal study from January 2016 to October 2016 was carried out at 10 sites of interest in Saint-Louis city. Frequency of sampling was monthly, and number of samples collected was 100 over 10 months (Figure 1). At time of each sampling, main physico-chemical parameters of the environment were recorded with equipment (portable conductimeter Sension+ EC5 HACH®): temperature, salinity derived from conductivity, pH. Filtration was carried out with a 0.22 μm membrane for 200 ml per sample. Membrane was then soaked in 10 ml of PBS buffer solution for 30 minutes. Then, 2 ml of this buffer solution was added to 18 ml of enrichment solution (alkaline peptoned water at pH=8.6).

Bacterial sample cultures from environmental samples were carried out on TCBS and on ChromAgar *Vibrio*. For biochemical characters, suspicious colonies (yellow or green colonies on TCBS) was used to do oxidase test and mini-identification gallery (glucose, lactose, citrate, gas). Because concentration of these bacteria in environmental samples may be low, it was also decided to investigate their presence using molecular markers by Polymerase Chain Reaction (PCR) method. Molecular analyzes were carried from January 16 to 26, 2017 at Pasteur Institute in Dakar on DNA extracts from APW solution and suspicious colonies from environment, respectively. For each sample, initial DNA concentration was measured with Nanodrop equipment and dilutions were made with physiological water for PCR to have a final concentration of 100 ng/μl. Multiplex PCR was first performed according to the protocol described by Kim *et al*. (2015) which uses primers for detecting Vibrio genus (VG) and following species: *Vibrio cholerae* (Vc), *Vibrio parahaemolyticus* (Vp), *Vibrio mimicus* (Vm), *Vibrio alginolyticus* (Va), *Vibrio vulnificus* (Vv) [14].

The volume of reaction was 25 µl of DNA dilution with an initial denaturation phase for 5 minutes by 94°C. Following by denaturation at 94°C for 30 seconds, then 25 cycles each including denaturation at 94°C for 30 seconds, following by hybridization at 60°C for 30 seconds and elongation at 72°C for 30 seconds. A final elongation at 72°C for 10 minutes ends reaction and storage can be made at 4°C. Migration of PCR products was carried by electrophoresis on 3% agarose gel prepared with ethidium bromide for revelation in a buffer of Tris acetate EDTA 0.5X. Different bands of gel was visualized at 220V with an intensity of 300mA under UV radiation. On samples with suspected *V. cholerae* positivity, simplex PCR was performed to confirm positivity. PCR, migration and visualization conditions are identical to those described above. Finally, different toxins were searched by PCR: the Cholera Toxin subunit A (CtxA) for samples positive for *V. cholerae* [15] and TRH and TDH toxins for samples positive for V. parahaemolyticus using specific primers [16].

**Sample size:** from the clinical surveillance among patients, 43 stool samples were received in the laboratory. From the environment, a total of 268 samples were analyzed by PCR and distributed as follows: 90 APW samples corresponding to 1 sample at each passage for each site, and 178 suspicious colonies isolated from TCBS and ChromAgar.

**Ethical consideration:** as this study involved human participants, human material, or medical data, we obtained the approval from the National Health Research Ethics Committee of Senegal (#284/MSAS/DPRS/DR, May 13, 2013). The patients were informed by the practitioner orally and received an explanatory letter about the current study detailing how the data and sample will be used in the respect of principles expressed in the Declaration of Helsinki. Stool samples were taken from orally consented patients.

## Results

**Clinical surveillance:** from the clinical surveillance among patients, 43 stool samples were received in the laboratory. Bacterial cultures were positive in 6% of cases but no strain of *Vibrio cholera* or other vibrios was isolated. Pathogenic bacteria identified were *Escherichia coli, Salmonella spp, Pseudomonas aeruginosa* with no further investigation was carried out. Some samples showed parasites such as *Entamoeba histolytica* and *Entamoeba coli* cysts, Lumbricoid Ascaris eggs. In two children, presence of rotaviruses responsible for gastroenteritis has been noted.

**Figure 2:**
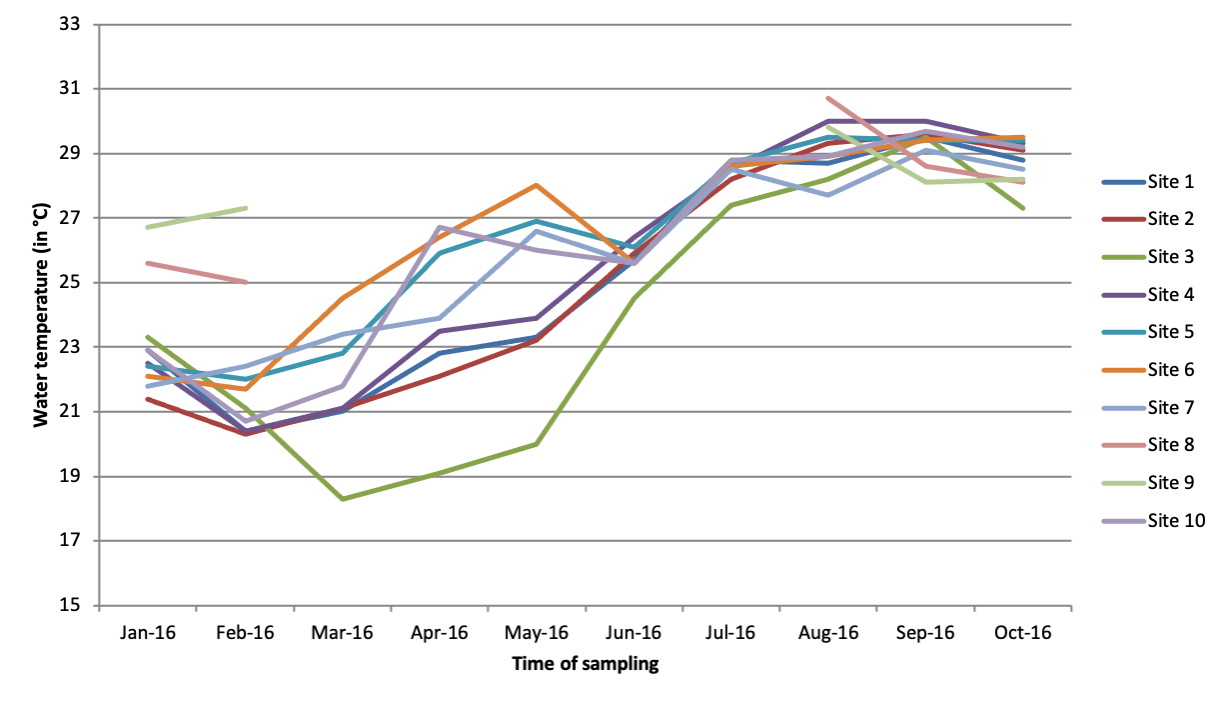
evolution of water temperature at sampling points in Saint-Louis city between January and October 2016

**Figure 3:**
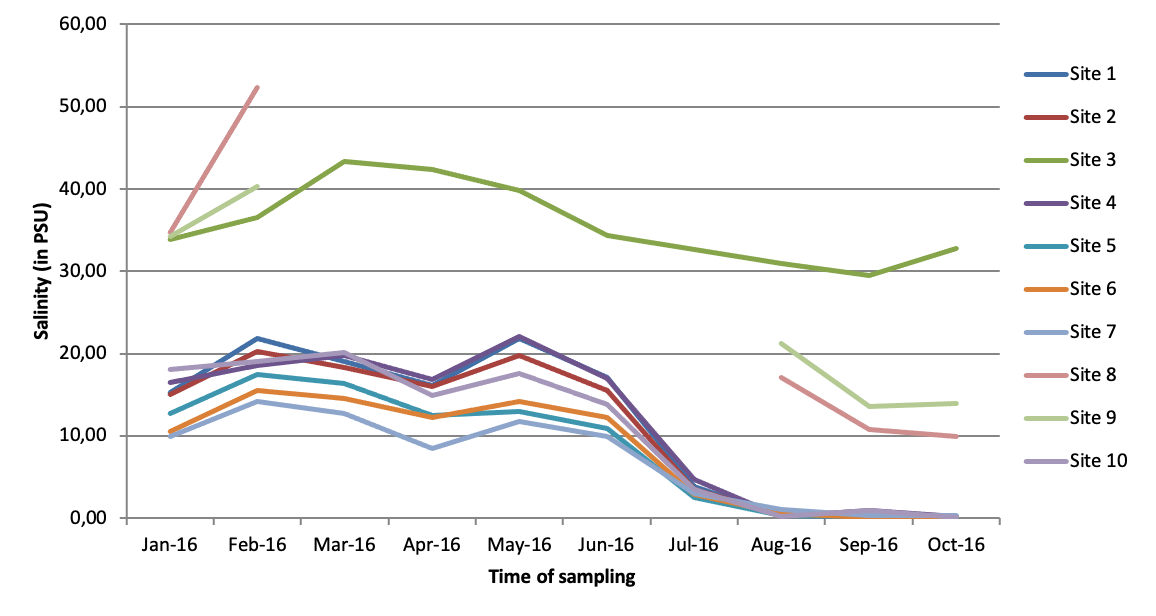
evolution of the water salinity at the sampling points in Saint-Louis city between January and October 2016

**Environmental surveillance:** from the environmental surveillance, on the selected 10 sites, 2 (sites # 8 and # 9) could not be sampled for five consecutive months because the river water had withdrawn during the dry season. These result in 90 water samples instead of the 100 planned. Water temperature fluctuated between 18°C and 31°C (Figure 2). With exception of site # 3, which has lowest temperatures and corresponds to water withdrawal point on coast, Guet Ndar (Chaumière), all of temperatures varied similarly according to a unimodal seasonality. Regarding salinity, it fluctuates between 0 and 50 PSU (Figure 3). Except for sites # 3, 8 and 9 which have higher salinities, and which correspond to the water withdrawal points on the coast (# 3) or in a shallow lagoon arm (# 8, 9), all salinities vary similarly according to a unimodal seasonality.

**Culture results:** on TCBS, suspect colonies subjected to microscopic examination revealed Gram-negative mobile bacilli. Biochemical characters were positive for oxidase and glucose, and negative for lactose, mannitol, hydrogen sulfide, urease, indole and tryptophan deaminase. On ChromAgar Vibrio dishes, identification of species was based by color of colony (uncolor for *V. alginolyticus*; blue for *V. cholera* or *V. vulnificus*; purple for *V. parahaemolyticus*).

**Figure 4:**
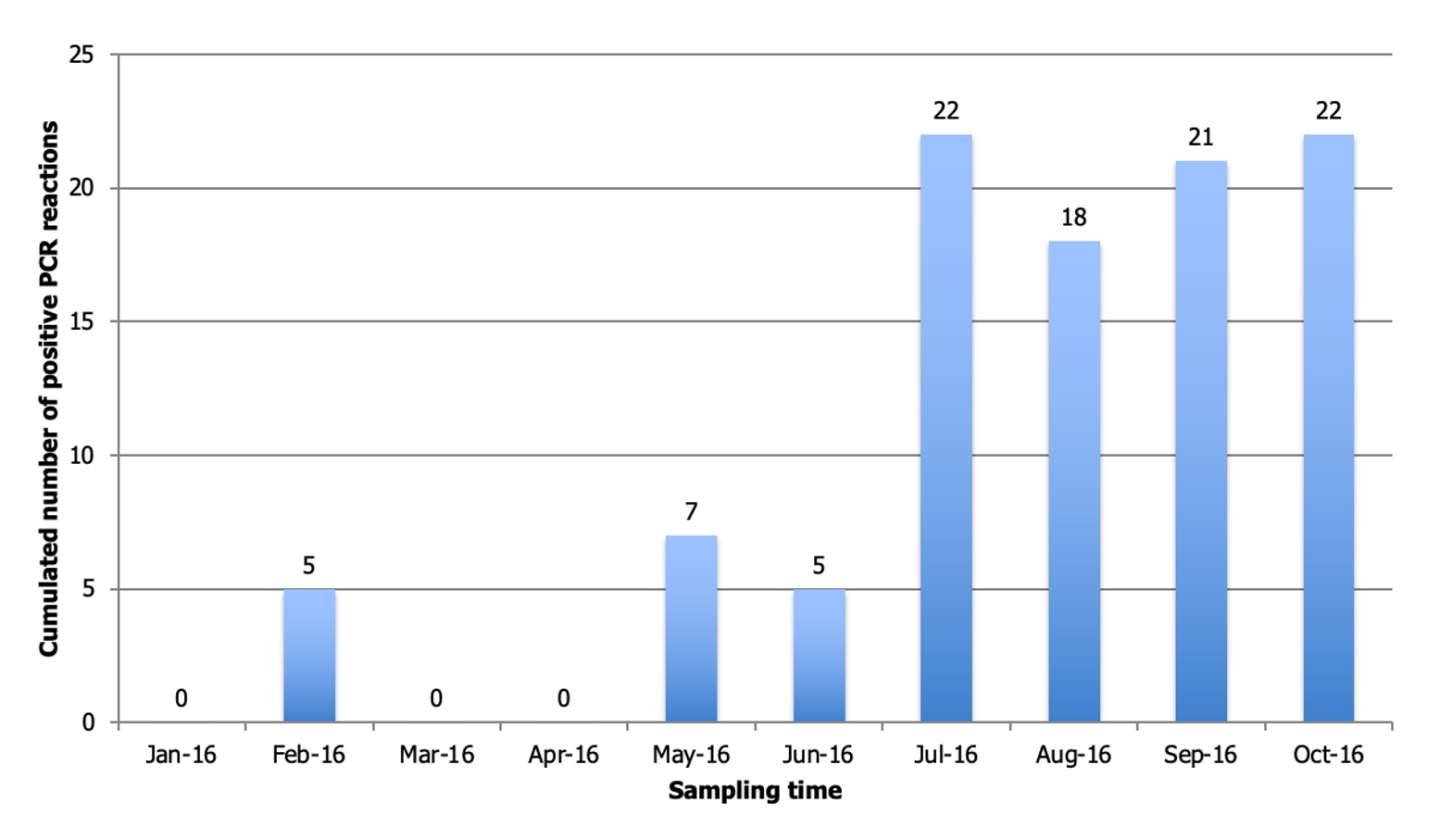
cumulative evolution of positive PCR markers on all sites according to time

**PCR results:** molecular tests were carried out on APW solutions and suspicious colonies isolated from ChromAgar. On 90 APW samples, markers of following species were positive in PCR: Vibrio sp. (n = 43), *Vibrio cholera* (n=27), *Vibrio mimicus* (n = 16), *Vibrio parahaemolyticus* (n = 8), *Vibrio alginolyticus* (n=4) and *Vibrio vulnificus* (n = 2). Cumulative number of positive PCR markers all combined from APW across all sites as a function of time shows a clear seasonality with highest number of positive markers from May to October 2016. Except for the month of February when five samples were positive for the marker of the genus *Vibrio sp*. on some sites (# 1, 3, 4, 5, 6); detection of *Vibrio* is effective during months when temperature is highest, in particular from July to October, and salinity is the lowest (Figure 4). Multiplex PCR, carried on the 178 colonies isolated from enrichments media, showed a majority of *V. parahaemolyticus* (n = 40) and *V. cholera* non-O1 / O139 (n = 35). Following species were confirmed by simplex PCR: *V. mimicus* (n = 19), *V. alginolyticus* (n = 11) and *V. vulnificus* (n = 1). Among 40 strains of *Vibrio parahaemolyticus*, 6 carried TRH gene, three of which simultaneously expressed virulence gene TDH.

## Discussion

Presence of natural populations of Vibrionaceae in the aquatic environment of Saint-Louis city was confirmed by this study. Among identified species, those potentially implicated in human infections are *Vibrio cholera* non-O1 / non-O139, *V. parahaemolyticus* TRh and/or TDH positives, *Vibrio vulnificus*, and *Vibrio alginolyticus*. None of the sample were positive for toxigenic *V. cholera* O1 or O139.

*Vibrio parahaemolyticus* seems to be most studied in the aquatic environment in European countries and United States [17,18]. In Spanish study, *V. parahaemolyticus* expressing TDH and / or TRH were isolated from patients who consumed fresh oysters or bivalves caught from Atlantic coast [17,19]. In United States, *V. parahaemolyticus* was one of 3 commonly reported Vibrio and had become the main agent of human gastroenteritis associated with consumption of seafood. But most of the hospitalizations and deaths were due to *V. vulnificus* infection [18]. Explanation may be linked to fact that *V. parahaemolyticus* is found in a commensal situation with crustaceans and oysters in an aquatic environment [18,20]. Seasonal detection of Vibrios was very marked and followed a unimodal distribution with optimum temperature of warmest water and lowest salinity.

Cumulative number of positive PCR markers across all sites according of time shows a clear seasonality with highest number of positive markers from May to October 2016. Presence of Vibrio is correlated with factors like temperature and salinity level. Thus, detection of Vibrio was effective during months when temperature was higher, especially from July to October, and when salinity was lowest except for the month of February when five samples were positive for genus Vibrio sp. marker in some sites (# 1, 3, 4, 5, 6). This seasonal trend was consistent with that described in literature with an optimal habitat for these *Vibrionaceae* species for temperatures above 20°C and a salinity between 0 and 14 PSU. According to Raghunanth (2014), pathogenicity of *V. parahaemolyticus* was more marked when oxygen concentration was low and according to level of organic components in aquatic environment [21]. The link between contamination of raw mollusks by *V. parahaemolyticus* and water temperature was already reported, especially in spring and summer. Monitoring risk of emergence of disease in humans is an important aspect of prevention. Consequently, use of space remote sensing had constituted a major advance by providing a variety of information on monitoring terrestrial and oceanic environment, particularly by measuring abiotic and biotic factors [22].

Studies have shown highest frequency of Vibrio isolation during hot period associated with optimal salinity values between 0.2 and 2‰ [23]. Other factors such as those affecting height of sea level or physico-chemical characteristics of rivers were cited as having an importance in emergence of cholera [24]. Current climatic changes associated with shift in precipitation and numerous floods would also promote the spread of Vibrio.

However, even if these floods had never been a factor in epidemic explosion of cholera in Senegal, this was not case for the 2005 epidemic where it was shown influence of intense precipitation on transmission of the disease during an ongoing outbreak in populated areas [25]. In our study, six strains of *V. parahaemolyticus* were positive for TRH toxin gene and 3 expressed simultaneously TDH gene what constitutes a major result as first description of such strains in Senegal [26]. Indeed, pathogenic power of this bacterium is linked to presence of two hemolysins, a direct thermostable hemolysin (thermostable direct hemolysin or TDH) and a hemolysin related to direct thermostable hemolysin (tdh-related hemolysin TRH).

According to published data, virtually all isolates of *V. parahaemolyticus* associated with gastroenteritis produce one and / or other of the two hemolysins [16]. Differences were noted in detection of toxins from environmental strains. In densely populated areas of South Carolina and Georgia coasts, low levels of TDH and TRH were detected in environmental strains of V. parahaemolyticus [27]. In contrast, 52% of environmental strains from intensive shrimp mariculture sites on Pacific coast of Mexico carried TDH and / or TRH [28].

Detection of TDH at high levels in environmental strains has also been reported in United States [29]. Until now and with means used in our study, no strain of toxigenic *Vibrio cholerae* O1 or O139, responsible for cholera pandemics, had been isolated from environment in Senegal while no cholera outbreak was ongoing. The fact remains that presence of many natural populations of very seasonally marked Vibrionaceae reveals that aquatic environment of Saint-Louis proves to be a favorable habitat for these bacteria. It is not excluded that environment of Saint-Louis city could play an important role in transmission of cholera in an epidemic situation, particularly from months of May to October.

The study however has some limitations worth mentioning. First, the number of patients screened is low and below the expectation. It would have been better to expand the recruitment of patients to other community-based medical facilities to increase both the robustness of the study and the geographical coverage. Second, the limitation of the available funds limited the study to the detection of presence or absence as the quantification for toxigenic *Vibrio cholera* is determinant as it is a dose dependent disease. The one-year study is also a limitation compared to a multi-year study that would have been more informative.

## Conclusion

This study confirmed the presence of natural populations of *Vibrionaceae*, including those involved in human infections. The concerned species are *Vibrio cholera* non-O1 / non-O139, *V. parahaemolyticus* carrying the virulence genes TRH and / or TDH, *V. vulnificus*, and *V. alginolyticus*. The aquatic environment of Saint-Louis is proving to be a favorable habitat for these bacteria. With a strong fishing activity of fishery products, it is not excluded that it can play an important role in the transmission of cholera if an epidemic were to spread among the population.

### What is known about this topic

- *We already know that Vibrio sp. natural habitat conditions and presence of certain species in Senegal*.

### What this study adds

- *This is the first publication of systematic investigation of Vibrio sp. patient in a non-epidemic period in Senegal;*
- *The confirmation of the presence of toxigenic Vibrio sp. strains (V. parahaemolyticus TRH and/or TDH positive, V. vulnificus, V. alginolyticus) and the absence of pandemic toxigenic Vibrio cholerae O1 or O139 in the water environment in Saint-Louis in Senegal*.
